# MOSOF with NDCI: A Cross-Subsystem Evaluation of an Aircraft for an Airline Case Scenario

**DOI:** 10.3390/s26010160

**Published:** 2025-12-25

**Authors:** Burak Suslu, Fakhre Ali, Ian K. Jennions

**Affiliations:** Integrated Vehicle Health Management Centre, Cranfield University, Cranfield MK43 0AL, UK

**Keywords:** multi-objective optimisation, NDCI, mRMR, sensor selection, MOSOF, aircraft, ECS, engine, airlines, IVHM

## Abstract

Designing cost-effective, reliable diagnostic sensor suites for complex assets remains challenging due to conflicting objectives across stakeholders. A holistic framework that integrates the Normalised Diagnostic Contribution Index (NDCI)—which scores sensors by separation power, severity sensitivity, and uniqueness—with a Multi-Objective Sensor Optimisation Framework (MOSOF) is presented. Using a high-fidelity virtual aircraft model coupling engine, fuel, electrical power system (EPS), and environmental control system (ECS), NDCI against minimum Redundancy-maximum Relevance (mRMR) is benchmarked under a rigorous nested cross-validation protocol. Across subsystems, NDCI yields more compact suites and higher diagnostic accuracy, notably for engine (88.6% vs. 69.0%) and ECS (67.7% vs. 52.0%). Then, a multi-objective optimisation reflecting an airline use-case (diagnostic performance, cost, reliability, and benefit-to-cost) is executed, identifying a practical Pareto-optimal ‘knee’ solution comprising 12–14 sensors. The recommended suite delivers a normalised performance of ≈0.69 at ≈USD36k with ≈145 kh MTBF, balancing the cross-subsystem information value with implementation constraints. The NDCI-MOSOF workflow provides a transparent, reproducible pathway from raw multi-sensor data to stakeholder-aware design decisions, and constitutes transferable evidence for model-based safety and certification processes in Integrated Vehicle Health Management (IVHM). The limitations (simulation bias, cost/MTBF estimates), validation on rigs or in-service fleets, and extensions to prognostics objectives are discussed.

## 1. Introduction

The advancement of Integrated Vehicle Health Management (IVHM) is central to enhancing the safety, reliability, and operational efficiency of modern complex assets such as aircraft. Modern aircraft have evolved from collections of discrete components into highly integrated systems-of-systems, where mechanical, thermal, electrical, and computational subsystems are deeply interconnected. A fundamental challenge in designing effective IVHM systems lies in the strategic selection of sensors [1]. The conventional approach of instrumenting systems primarily for control is often insufficient for comprehensive diagnostics, as the subtle signatures of incipient faults can be easily missed. Faults in highly integrated systems rarely manifest in isolation. For instance, a malfunction in an engine’s bleed air system can propagate, causing performance shifts in the Environmental Control System (ECS) and load fluctuations in the Electrical Power System (EPS) [2]. An optimal sensor network must therefore possess the capability to capture these distributed, cross-subsystem effects to enable accurate fault isolation [3,4].

The process of selecting this optimal sensor set is a complex dimensionality reduction task. While generic feature selection algorithms, such as Minimum Redundancy–Maximum Relevance (mRMR) [5], are widely used, they have inherent limitations in the context of engineering diagnostics [6]. Such methods typically rely on statistical relevance and redundancy criteria, which do not fully capture a sensor’s true diagnostic utility. They may fail to differentiate between faults of varying severity and can discard correlated sensors that, despite statistical similarity, measure distinct physical phenomena crucial for robust diagnosis. This recognition led to foundational work establishing the need for domain-aware metrics that evaluate sensors based on their contribution to diagnostic objectives [1].

This paper builds directly upon that foundation. The previous research first conceptualised the limitations of generic methods and proposed a diagnostically-focused evaluation framework. Then, the Normalised Diagnostic Contribution Index (NDCI) and its initial application within a Multi-Objective Sensor Optimisation Framework (MOSOF) on a single aircraft subsystem are introduced [1]. The present study provides a comprehensive, cross-subsystem validation and refinement of this integrated NDCI-MOSOF methodology.

Sensor selection is intrinsically a multi-objective problem, requiring concurrent optimisation of often-conflicting goals. Stakeholders, including operators, manufacturers, and maintenance providers, must navigate the trade-space between maximising diagnostic coverage and minimising factors such as instrumentation cost, weight, and reliability penalties [7,8]. Single-objective optimisation cannot adequately address this complex decision-making landscape. Therefore, the MOSOF approach leverages a multi-objective genetic algorithm to explore this trade-space, identifying a set of non-dominated, or Pareto-optimal, solutions [9,10]. This methodology provides decision-makers with a portfolio of optimised sensor suites, each representing a different balance of priorities. This aligns with modern aerospace development guidelines, like ARP4754B [11], which advocate for systematic, model-based processes to substantiate safety and operational requirements [12].

Navigating this complex trade-space is a central challenge in IVHM design.

The trade-offs involved are significant and have direct operational consequences. The true cost of an aerospace-grade sensor extends far beyond its unit manufacturing price; it includes substantial overheads for certification, material traceability, regulatory documentation, and lifecycle support infrastructure [13]. It is not uncommon for a sensor with a manufacturing cost of EUR500 to have a market price of EUR5000 once these factors are included [13]. Similarly, weight is a critical consideration in aircraft design. Every kilogram of mass added to an airframe translates into significant lifecycle fuel costs, with industry estimates placing the cost of weight between USD1500 and USD2000 per kilogram over the aircraft’s operational life [14]. An optimised sensor suite that saves even a few kilograms can therefore yield millions of dollars in operational savings for airlines, as the cost of weight between USD100 and USD1000/kg has been confirmed to be reasonable in other studies [15]. Finally, every component added to a system introduces a new potential point of failure, which can decrease the overall system’s Mean Time Between Failures (MTBF). This reliability penalty must be carefully weighed against the diagnostic benefits the sensor provides.

This multi-objective landscape is further complicated by the divergent priorities of the key stakeholders involved in an aircraft’s lifecycle. Original Equipment Manufacturers (OEMs) are often focused on minimising manufacturing costs, simplifying the design to streamline certification against standards like ARP4754B [11], and ensuring compliance with airworthiness directives. Airlines and other operators, conversely, prioritise operational continuity, seeking to maximise fleet availability, reduce lifecycle maintenance costs, and achieve the highest possible benefit-to-cost ratio for any new technology they adopt. Finally, Maintenance, Repair, and Overhaul (MRO) providers value sensor systems that enable rapid and unambiguous fault isolation, as this directly reduces aircraft-on-ground (AOG) time and expedites the repair process. An optimal sensor selection framework must be capable of reconciling these varied and often competing interests.

This physical reality of fault propagation demands a holistic, system-of-systems approach to sensor placement. A sensor suite that has been optimised for a single subsystem in isolation is likely to be globally suboptimal and may even be unsafe. Traditional design processes, which often involve separate component teams submitting lists of desired measurements for their specific subsystems, create a siloed view of the aircraft’s health [16]. This approach inevitably creates a “diagnostic blind spot” at the interfaces between subsystems. For instance, an optimisation focused solely on the engine might discard a sensor that, while only moderately useful for diagnosing engine-internal faults, is critically important for observing the downstream effects of a bleed air anomaly on the ECS. Conversely, an ECS-focused optimisation would have no logical basis for selecting a sensor located on the engine, even if that sensor provides the earliest and clearest indication of an impending bleed air problem. Only a unified optimisation framework that considers all candidate sensors and all potential fault modes across the entire platform simultaneously can “see” into this blind spot. Such a framework can identify and select sensors based on their cross-subsystem diagnostic value, a capability that is not merely an improvement but a necessary condition for achieving comprehensive fault coverage in modern, integrated aircraft.

This study makes the following contributions to help overcome this bottleneck problem in the diagnostics domain: (i) It benchmarks the refined NDCI against mRMR across four critical aircraft subsystems using a robust nested cross-validation protocol for reliable performance estimation. (ii) It demonstrates the framework’s ability to identify and leverage cross-subsystem synergies for improved diagnostics. (iii) It applies the complete MOSOF to a realistic airline case study, identifying a practical, cost-effective sensor suite from the Pareto-optimal front.

The remainder of this paper is structured as follows: Section 2 details the materials and methods, including the mathematical derivation of the NDCI metric, the problem formulation, and the Virtual Aircraft Model (VAM) setup. Section 3 presents the subsystem-level analysis and ranking comparisons. Section 4 details the platform-level MOSOF optimisation and the resulting Pareto analysis. Finally, Section 5 discusses the implications for stakeholders, followed by conclusions in Section 6.

## 2. Materials and Methods

This section formalises how the NDCI–MOSOF pipeline, from a single-subsystem study to a platform-level optimisation across four interacting aircraft subsystems—engine, fuel, electrical power system (EPS), and environmental control system (ECS)—is extended. The goal is to select sensor suites that (i) detect degradation, (ii) isolate fault modes, and (iii) respect stakeholder constraints (OEM, Airline, MRO) on cost, reliability/efficiency, and compatibility. The modelling and optimisation machinery, faithful to the previous work and its ECS validation, is retained, and the data interfaces, metrics, and constraints that allow cross-subsystem synergies and scenario-wise coverage are generalised.

The process started by assembling a platform-level data matrix from the four subsystems. Each record is associated with a scenario (healthy or a specific fault mode at a given severity). Pre-processing unifies sampling and units, removes outliers and near-constant channels, and applies z-score normalisation/standardisation. Raw simulation data are pre-processed to remove constant or nearly constant channels. A channel is discarded if its variance after normalisation is less than ${1\times \;10}^{-6}$ or if it fails to deviate from the baseline by more than a noise floor during any fault condition.

A mapping table links every sensor to its owning subsystem and to the set of fault modes it can potentially inform. This yields the scenario sheets used throughout the pipeline.

Feature ranking then proceeds along two complementary streams. The first is the NDCI stream, where three ingredients: (1) Separability (SP): how well a sensor separates the scenario from competing classes; (2) Sensitivity: monotonic change with degradation severity; and (3) Uniqueness: non-redundancy with respect to the current candidate set are computed per scenario. These are normalised and combined into an NDCI matrix, which is aggregated across scenarios to obtain an overall ranking. In parallel, a baseline mRMR stream produces a global relevance–redundancy ranking using the same labels. The dual stream provides a robustness check and an independent baseline for comparison.

Because a single global ranking can under-serve rare but safety-critical cases, next, the coverage-based minimal subsets per scenario are derived. For each scenario, the smallest subset achieving a pre-specified fraction (e.g., ≥95%) of the best achievable scenario-NDCI is retained. These subsets are computed separately for the NDCI and mRMR streams, and their union is taken across scenarios, a simple technique that preserves coverage without increasing the set size.

The resulting candidate pools serve as the seed for the MOSOF, which is solved using a multi-objective genetic algorithm (MOGA) [17]. The decision variable encodes exactly k sensors per policy. Objectives include performance (NDCI and/or classifier coverage/accuracy), cost (Σ cost), reliability/efficiency (e.g., Σ (MTBF – 1)^−1^ or Σ efficiency loss), and a stakeholder criterion (OEM compatibility or Airline/MRO benefit-to-cost). The output of the MOGA process is not a single “optimal” solution but rather a set of non-dominated, or Pareto-optimal, solutions. A sensor suite is defined as Pareto-optimal if no single objective (such as diagnostic performance) can be improved without simultaneously degrading at least one other objective (such as cost or reliability) [18]. This collection of non-dominated solutions forms the Pareto front, which represents the boundary of best-achievable designs. This Pareto front serves as a powerful decision-support tool. It provides stakeholders with a transparent portfolio of optimised sensor suites, each representing a different, quantitatively evaluated trade-off between performance, cost, reliability, and other key objectives. Hard constraints enforce a budget cap, a minimum summed NDCI, a minimum average diagnostic performance, and a minimum reliability/efficiency. The outcome is a Pareto front from which stakeholder-specific choices can be read off.

Finally, the selected suites, using repeated/nested cross-validation with lightweight classifiers (bagged decision tree), are validated and generalised. The detection (balanced accuracy) and isolation (per-fault mode accuracy) are reported, along with confusion matrices, stepwise feature histories, and low-dimensional separability visuals for interpretability.

**Figure 1:**
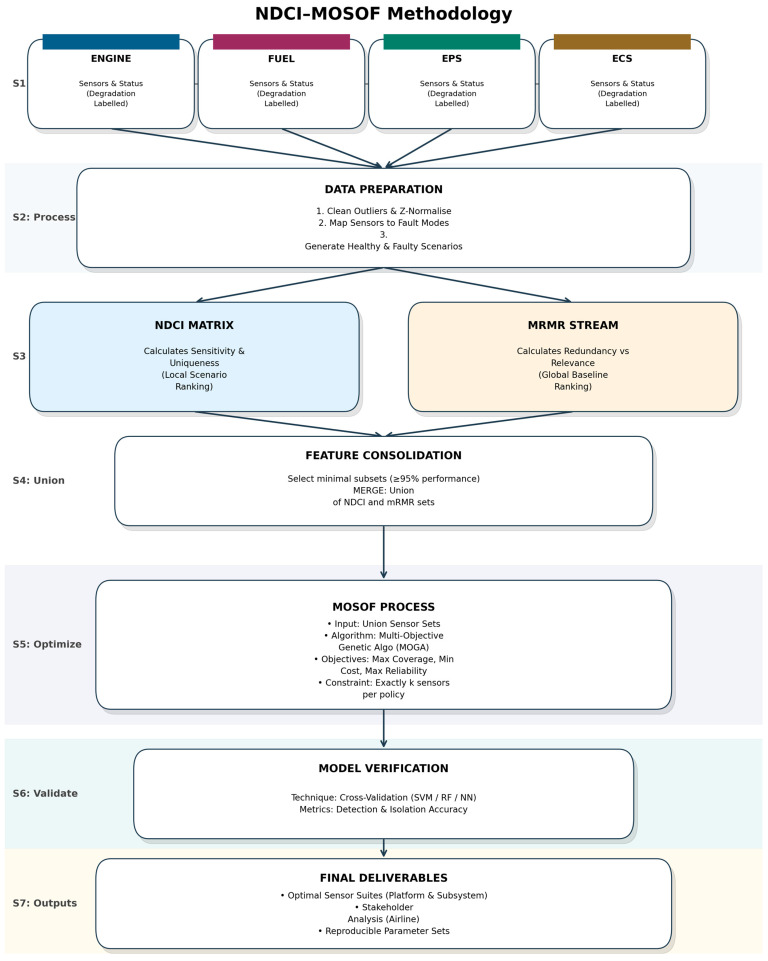
NDCI–MOSOF methodology for multi-subsystem aircraft diagnostics.

Figure 1 summarises the end-to-end workflow, from data assembly and the dual metric streams to coverage-aware pooling, MOSOF/MOGA optimisation, and validation.

S1-Sources: Multi-sensor streams from four interacting subsystems—engine, fuel, electrical power system (EPS), and environmental control system (ECS)—with scenario labels covering healthy and fault-mode/severity conditions; dashed connectors indicate cross-subsystem couplings. S2-Process: Platform-level assembly; removal of outliers and near-constant channels; temporal alignment and z-normalisation; mapping of sensors to subsystems and fault modes; construction of scenario sheets. S3-Dual metrics: (i) NDCI stream computes, per scenario, separability (SP), sensitivity to degradation, and class-uniqueness to form an NDCI matrix and aggregate ranking across scenarios; (ii) *mRMR baseline* provides a relevance–redundancy ranking using the same scenario/severity labels. S4- Union: For each scenario, minimal sensor subsets that retain ≥95% of the best-scenario NDCI are found separately for NDCI and mRMR; unions across scenarios preserve coverage against case variation. S5-Optimisation: A multi-objective genetic algorithm (MOGA) searches exactly k-sensor policies under constraints (budget cap, minimum ΣNDCI, minimum average detection/isolation performance, minimum reliability/efficiency). Objectives span (f_1_) performance (NDCI and/or classifier coverage/accuracy), (f_2_) cost, (f_3_) reliability/efficiency, and (f_4_) a stakeholder criterion (OEM compatibility or Airline/MRO benefit-to-cost). The panel includes a schematic Pareto front for emphasis. S6-Validation and generalisation: Repeated/nested cross-validation with SVM, Random Forest, and small neural nets; metrics include balanced accuracy for detection and per-fault-mode isolation; artefacts include confusion matrices and low-dimensional separability visuals. S7-Outputs: Pareto fronts and recommended suites at platform and subsystem level, stakeholder-specific selections (OEM/Airline/MRO), cross-subsystem synergies, and packaged code/parameters for reproducibility.

### 2.1. Normalised Diagnostic Contribution Index (NDCI) vs. mRMR

The NDCI is a composite metric specifically engineered to quantify a sensor’s diagnostic value, moving beyond generic statistical measures. As established in [1], the NDCI integrates three normalised components that reflect key diagnostic properties:

- Separation Power (SP): This component quantifies the magnitude of a sensor’s response to a fault relative to its baseline noise. It is calculated as the mean absolute deviation of the sensor signal from its healthy baseline across all fault conditions, normalised by the dynamic range observed during healthy operation.
- Severity Sensitivity (S): This component measures how well a sensor’s response tracks the progression of a fault. It is computed by normalising the sensor’s absolute deviation by a factor representing the fault’s severity, rewarding sensors that show a monotonic response as degradation deepens.
- Uniqueness (U): This component promotes informational diversity within the sensor suite. To avoid selecting clusters of redundant sensors, it penalises signals that are highly correlated with others. It is computed as one minus the average absolute Pearson correlation between a given sensor and all others under fault conditions. It is important to distinguish that the NDCI “Uniqueness” component penalises mathematical redundancy (high statistical correlation) rather than physical coupling. This ensures that the algorithm selects sensors along the propagation path of a fault rather than clustering multiple sensors at the single point of highest impact.

Each component (SP, S, and U) is scaled to a range and averaged to produce a single, unified NDCI score. A high-NDCI sensor, therefore, exhibits a clear and robust response to faults, tracks fault progression, and provides information that is distinct from other sensors in the network.

FM’s descriptions and the associated subsystems’ complete list are shown below in Table 1.

### 2.2. Data and Methods

This study utilises a high-fidelity Virtual Aircraft Model (VAM), which integrates coupled simulation models of four critical subsystems to capture realistic interactions. The data collected for this work only considers the ground-level runs, and the description of the sub-systems is as follows.

- Engine: The model chosen for the engine digital twin is the Pratt and Whitney JT9D open-source turbofan engine model provided by T-MATS software in MATLAB R2024a Simulink [18].
- Fuel System: A Simulink-based digital twin of a laboratory fuel rig, capable of simulating pump degradation, blockages, and other flow-related faults [2].
- Electrical Power System (EPS): A simulation model of the EPS, which models generator load dynamics with an Adaptive Neuro-Fuzzy Inference System for its diagnosis (ANFIS) [2].
- Environmental Control System (ECS): Proprietary models that simulate bleed air management and thermal performance of heat exchangers.

The integrated VAM platform allows for the simulation of cascading faults, providing a rich dataset for training and validating diagnostic algorithms [19]. Raw simulation data are pre-processed to remove constant or nearly constant channels, and the remaining signals are then z-scored. An initial redundancy check removes one sensor from any pair with a Pearson correlation greater than 0.995.

To rigorously compare the performance of NDCI and mRMR, a nested, aggregated cross-validation protocol was employed. This method prevents information leakage from the test set into the feature selection process, yielding more reliable and less optimistic performance estimates. An outer loop partitions the data for training and testing. Within each training partition, an inner cross-validation loop is used to determine the optimal number of sensors (k) for a given ranking method. A classifier is then trained on the full training partition using the top k sensors and evaluated on the held-out test set. To account for class imbalance in the fault data, balanced accuracy is used as the primary performance metric.

#### Problem Formulation

The sensor selection process is formulated as a Multi-Objective Optimisation problem. Let X = {$x_{1}{,x}_{2},\;\ldots ,\;x_{N}$} be the set of N candidate sensors available across the platform. The decision vector S represents a selected subset of sensors of size k. The objective is to identify the set of non-dominated solutions (the Pareto frontier) that simultaneously optimise the following conflicting objectives:

- Maximise Diagnostic Performance ($f_{1}$): Defined as the aggregated classifier accuracy or the cumulative NDCI score of the selected suite:

(1) $$f_{1}\left(S\right)=Accuracy\;\left(S\right)+\sum_{x_{i}\;(S)}NDCI\;(x_{i})$$

- Minimise Cost ($f_{2}$): Defined as the sum of acquisition and integration costs for the selected sensors:

(2) $$f_{2}\left(S\right)=\sum_{x_{i}\;\left(S\right)}cost\;\left(x_{i}\right)$$

- Maximise Reliability ($f_{3}$): Defined as the harmonic Mean Time Between Failures (MTBF) of the suite, ensuring the system is not compromised by its weakest link:

(3) $$f_{3}\left(S\right)\;\frac{k}{\sum_{x_{i}(S)}^{1/}\left({MTBF}_{i}\right)}$$

The computational complexity of the framework consists of two phases: feature ranking and evolutionary optimisation. The NDCI calculation involves pairwise correlation checks to determine uniqueness, resulting in a complexity of O(N^2^) where N is the total number of candidate sensors. The subsequent MOSOF phase employs the NSGA-II algorithm to search for optimal subsets of size k. The primary computational bottleneck in this phase is not the genetic operators, but the fitness function evaluation, which requires training and validating a classifier for each candidate solution in the population. To mitigate this, lightweight classifiers (Bagged Trees) were selected to ensure the search remains computationally feasible.

### 2.3. Classifier Evaluation

To decouple ranking effects from learner effects and to select a sensible default learner for deployment, an inner cross-validation (CV) search is performed within each outer-fold training split. The classifier catalogue comprises class-weighted Bagged Trees (Bag), SVM with RBF kernel (svmRBF), subspace k-nearest neighbours (subKNN), and Kernel Naïve Bayes (nbKernel); inverse-frequency misclassification costs address class imbalance for all learners. For a given ranking method and subsystem, features are added stepwise in the learned rank order, inner-CV balanced accuracy is recorded across steps, and a one-standard-error rule selects the number of k features. The learner is chosen by the inner-CV peak under the same rule; all steps use training partitions only to prevent information leakage into the outer test. The best performers in each subsystem for detection and isolation tasks are shown in Table 2.

Detection tasks across all subsystems are best served by class-weighted Bagged Trees. For isolation, Bag remains the preferred learner on Engine and ECS, svmRBF is preferred on EPS, and nbKernel is preferred on Fuel. These choices are consistent with subsystem-specific decision-boundary geometry and the diversity of available sensors; they are used as the default learners when reporting deployed confusion matrices and quantitative analysis.

## 3. Results

This section reports a two-step path from a platform-level recommendation to a subsystem run for comparison:

1. Platform symptom vectors.
  How each fault perturbs sensors across the subsystems is visualised by plotting normalised deviations from the healthy baseline and fault signatures are generated. These subsystem-level fault mode readings are used to generate platform-level plots, which show where a fault’s influence is concentrated and how it propagates into other subsystems. The platform level comparisons justify the subsequent design choices by revealing genuine cross-subsystem couplings, while also showing that a single platform-wide ranking is not robust for this dataset.
2. Subsystem-level, quantitatively comparable rankings and classifiers.
  Because a global ranking was unstable (dominated by a few high-variance channels and heterogeneous scaling), feature rankings and classifiers are evaluated per subsystem. This isolates method effects, prevents cross-subsystem leakage during training, and yields comparable, leakage-free performance estimates for engine, fuel, ECS, and EPS. The resulting candidate sensors and their measured diagnostic value are then handed to MOSOF (Section 4), which composes platform suites that mix sensors across subsystems under cost/reliability constraints.

**Figure 2:**
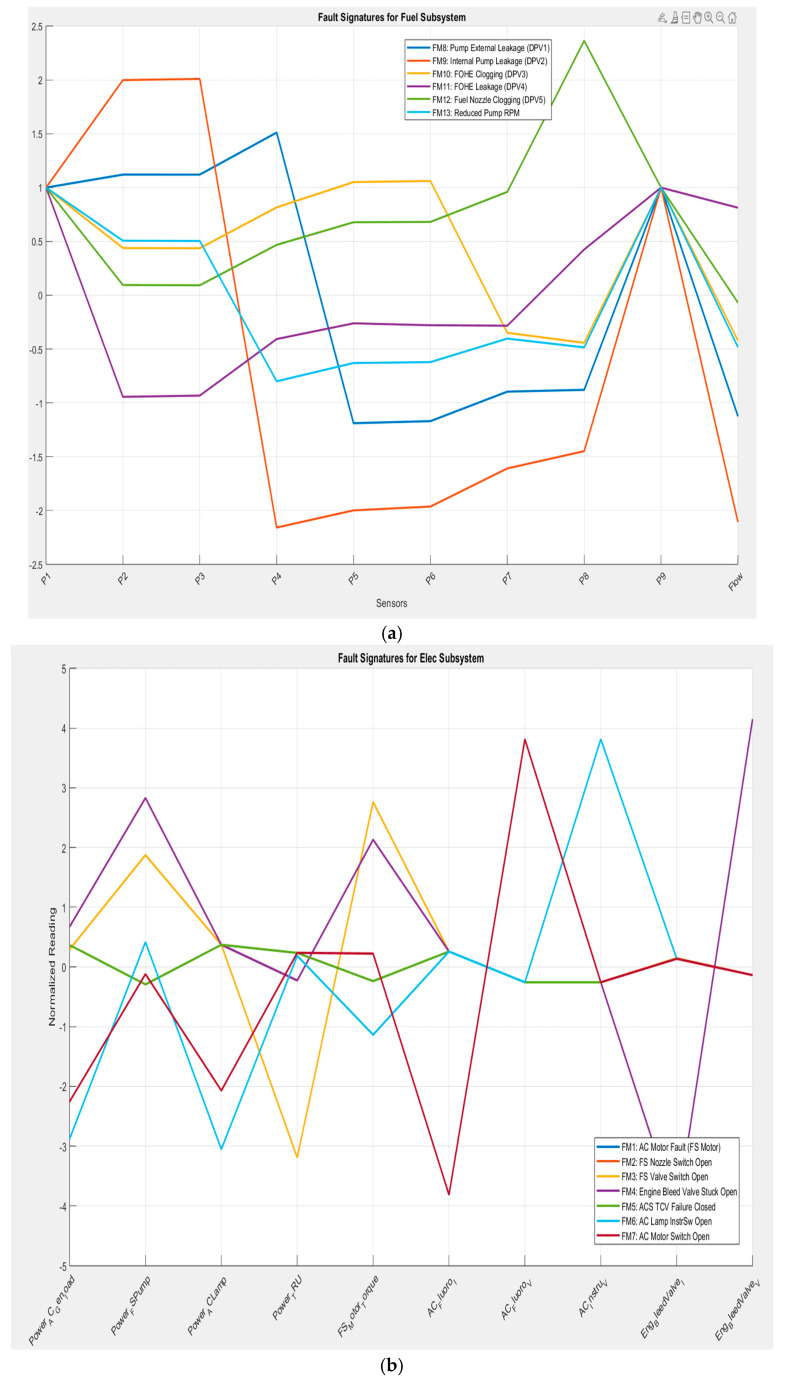
(**a**) Fuel fault signatures and (**b**) EPS fault signatures—example of system symptom vectors.

**Figure 3:**
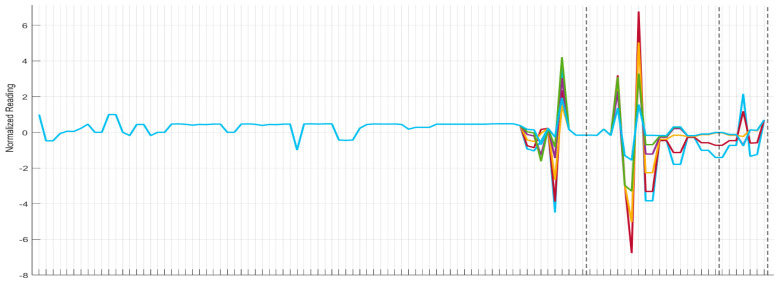
FM 5 severity sweep (0.1 incremental) across PSV (standardised sensors readings across the platform).

In short, the Figures at the start of this section present subsystem-level fault signatures (Figure 2) that are used in this work and the platform-level symptom vectors (Figure 3) for interpretation. Section 4 returns to the platform to optimise and select a cross-subsystem suite.

### 3.1. Cross-Subsystem Synergies and Feature Ranking

The platform-level “symptom vectors” that visualise how each fault mode perturbs sensors across subsystems are shown before presenting the quantitative, subsystem-level rankings. For a given fault and severity, each sensor reading is expressed as a normalised deviation from the healthy baseline; plotting these deviations as a vector reveals where the effect concentrates and how far it propagates into other subsystems.

Directly optimising a single platform-wide ranking with either NDCI or mRMR proved unstable and physically not sensible for this dataset (dominated by a few high-variance channels and heterogeneous scaling across subsystems). Therefore, the platform symptom vectors (PSV) are used for interpretation only and perform the quantitative comparisons per subsystem. The figures below establish the cross-subsystem context that motivates that design choice.

Figure 2a, the six fuel faults (FM8–FM13) produce distinct signatures over the pump pressures (P1–P7) and the flow channel. Pump-leakage and reduced-RPM faults show broad, moderate deviations across pressures, whereas Fuel Oil Heat Exchanger (FOHE)-related faults concentrate around the manifold taps.

In Figure 2b, EPS faults (FM1–FM7) generate characteristic responses on power-related channels (e.g., generator load, lamp/instrument buses). Notably, faults that involve bleed-air actuation (e.g., valve/motor issues) imprint on electrical measurements via load changes, illustrating coupling between electrical actuation and the pneumatic path. These two subsystems’ fault signatures and the FM5 PSV are presented as illustrative examples; the full figures can be reproducible from the code bundle [20].

FM5, represented in Figure 3, is an EPS fault named “ECS Temperature Control Valve Switch Open”. Curves show the normalised deviation across all sensors as the FM5 severity increases; vertical dashed lines mark subsystem boundaries. Excursions of the FM5 EPS fault grow non-linear in the ECS chain, and other electrical load channels exhibit similar responses. These patterns indicate how the severity of one fault mode affects the sensor readings of other subsystems. The informative content is highly localised to the Fuel subsystem, with limited propagation to other subsystems. The concentration of severity-dependent changes around these regions is the principal cross-subsystem synergy leveraged by MOSOF later.

Where the “macro effects” appear and how they shape the solution are derived from the PSV of each fault mode. The platform-level symptom-vector figures establish the macro pathways highlighted in the introduction, by originating each PSV of each FM:

- Fuel → engine. Fuel-system faults modulate flow and manifold pressures and, through combustion, alter engine temperature/enthalpy signatures. This is visible in the fuel and engine symptom-vector plots and reappears in the final suite via flow (fuel) and engine thermodynamic channels with high NDCI scores.
- Engine bleed-air → ECS thermal chain. Electrical FM5 (ACS TCV closed) drives strong excursions along the ECS heat-exchanger temperature chain and the engine bleed/mass-flow variables; the cross-subsystem effect distribution shows the largest bars on ECS thermal sensors with a concurrent spike on bleed-air mass-flow. NDCI prioritises these same regions and persists in multi-objective selection.
- Pneumatic/thermal → electrical load. Actuation and valve states couple back into electrical channels through load changes, explaining why some EPS power measurements enter the Pareto-efficient suites despite modest stand-alone separation.

**Figure 4:**
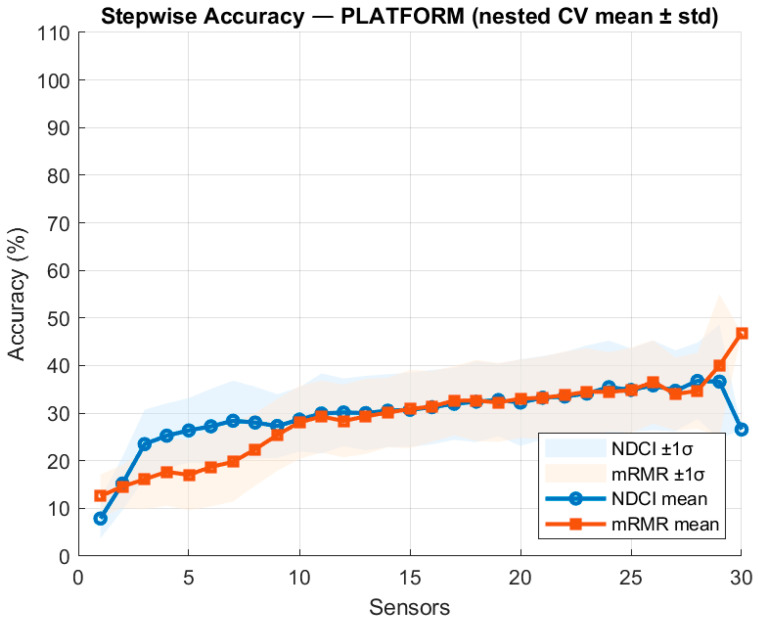
Stepwise accuracy for the platform-level evaluation.

These platform-level visuals for each FM’s effects on the sensor readings of other systems and their evaluation are based on the system interactions. The Platform-wide rankings did not yield sensible results for this dataset; however, the cross-subsystem effects are real and can be best exploited by building subsystem-level rankings and then allowing MOSOF to combine sensors across subsystems. The evaluation of the stepwise accuracy for platform-level results is shown below in Figure 4.

To test whether a single platform-wide ranking could be deployed directly, a global experiment pooled all candidate sensors across subsystems and evaluated NDCI and mRMR by adding features stepwise based on the technique’s ranking. The platform-level stepwise accuracy in Figure 4 shows a low early rise (≈20–25% by 3–5 sensors) followed by a broad plateau around 30–35% with wide fold-to-fold variability; additional sensors beyond ≈ 10 sensors yield only marginal accuracy gains. This behaviour indicates that a single global ranking does not yield reasonable results for this dataset; the heterogeneous scales and class distributions across subsystems diminish discriminability when features are mixed globally, yet the cross-subsystem effects are real, as established by the symptom-vector visuals above. Consequently, all quantitative ranking comparisons are performed per subsystem (Engine, Fuel, EPS, ECS) to obtain stable, leakage-free estimates.

### 3.2. Baseline vs. Nested Cross-Validation Performance

Baseline experiments were conducted using a simple five-fold cross-validation protocol on a dataset with limited samples, which included three degraded, faulty readings with varying severity levels, as well as a healthy reading for each fault mode. The results of the baseline runs yielded an optimistic outcome, given the limited number of readings, as shown in Table 3. Therefore, a more comprehensive technique, nested cross-validation, is applied to enhance the accuracy of the results. The nested cross-validation protocol:

- Goal: Obtain unbiased performance estimates while comparing ranking methods fairly.
- Outer loop (testing): Data are partitioned into outer training and test folds (stratified by fault mode). The test fold remains untouched until the final evaluation in that outer iteration.
- Train-only ranking: Within each outer training set, NDCI and the baselines (e.g., mRMR) are computed using training data only. Redundant sensors are pruned on training faults.
- Inner loop (model selection): On the training faults only, an inner K-fold loop evaluates a single, fixed classifier while adding sensors stepwise in the given rank order (top-1, top-2, …). At each step, the inner-CV mean ± std accuracy is recorded. The number of sensors, k, is chosen by a one-standard-error (SE) rule from this inner loop.
- Fit and test: Using the top-k sensors for that ranking, the whole outer training set is retrained on and predicts the held-out outer test faults.
- Repeats and aggregation: The entire process is repeated with different partitions; outer-test predictions are pooled to form the confusion matrices and summary metrics (balanced accuracy for detection/isolation).
- The descriptions of the figures:
  - Stepwise curves plot inner-CV mean ± std accuracy vs. the number of sensors added; this reflects the sample efficiency of each ranking.
  - Tables/summary bars report outer-test performance aggregated over repeats/folds; this reflects generalisation.
  - Confusion matrices aggregate outer-test predictions; this reveals which faults are confused in practice.

This protocol ensures that feature ranking, selection of k, and final testing are cleanly separated. All ranking methods are evaluated using the same classifier (class-weighted bagged decision tree ensemble), metrics, and splits, so differences arise from the ranking itself rather than from modelling choices.

As shown in Table 3, under these conditions, NDCI demonstrated a significant advantage over mRMR. For the Engine subsystem, NDCI achieved 92.6% balanced accuracy with only 9 sensors, compared to 77.8% for mRMR with 12 sensors. Similar gains were observed for the EPS (73.1% vs. 34.6%) and fuel (66.7% vs. 58.3%) subsystems.

To obtain more reliable performance estimates, we switched to a nested, aggregated cross-validation protocol with an increased sample size of around 40 readings per fault class. The results, summarised in Table 4, show an expected decrease in overall accuracy scores due to the more challenging and realistic validation scheme. However, the performance advantage of NDCI was largely preserved. For the engine, NDCI’s accuracy remained high at 88.6%, while mRMR’s performance dropped sharply to 69.0%. For the ECS, NDCI achieved 67.7% accuracy versus 52.0% for mRMR. The fuel subsystem remained a challenge for both methods, with mRMR showing a slight advantage (53.0% vs. 48.7%), an outcome attributed to the limited diversity of available sensor types for that subsystem. This aspect’s enhancement could be future work for the NDCI improvement.

The large gap between the simple baseline and the nested protocol is methodological. The baseline used a single pass cross-validation in which feature ranking and the choice of the number of sensors were not separated from evaluation; this allows information from the test fold to influence selection (optimistic bias under small samples). The nested protocol enforces separation and adds repeatability:

- Repeats/folds: 10 repeats; 5 outer folds for testing; 3 inner folds for model selection.
- Train/test split: within each outer fold, feature ranking uses training partitions only; held-out tests are never seen until the final evaluation.
- What is ranked: training-fault rows with a healthy baseline; redundant sensors pruned at |ρ| > 0.995 on training data.
- Learner: a class-weighted bagged decision-tree ensemble (identical across methods) with inverse-frequency class costs.
- Model selection: inner-CV stepwise curves determine k using a one-SE rule; only the top-k sensors are carried to the outer test.
- Metric alignment: stepwise panels show inner-CV accuracy (mean ± sd); outer-test summaries report balanced accuracy aggregated across repeats.

Under this protocol, the performance drops from the inflated baseline to values that agree with physical intuition and platform-level symptom vectors. The lesson is straightforward: without nesting, the evaluation overstates what a deployable system would achieve; with nesting, estimation variance tightens, and conclusions become defensible.

**Figure 5:**
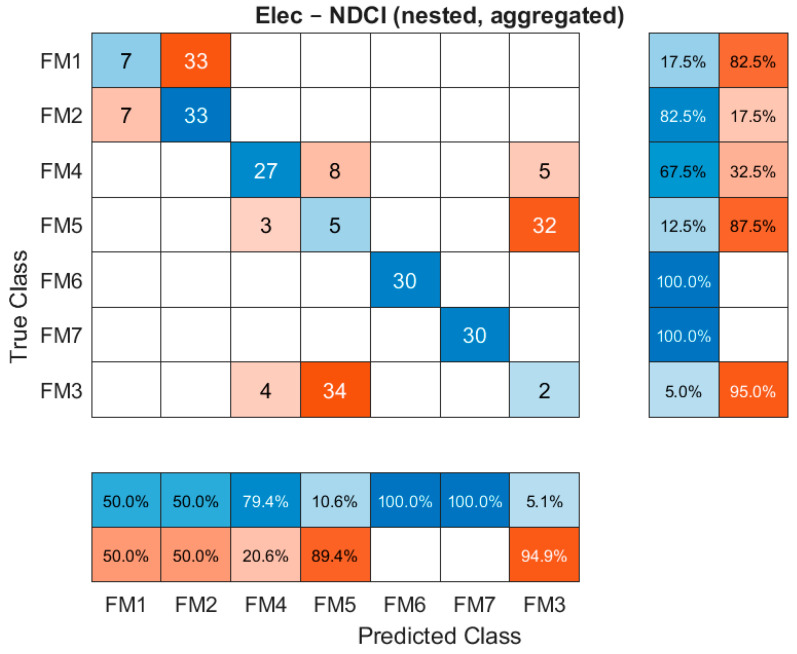
Confusion matrix for the EPS subsystem using the NDCI-selected suite (nested, aggregated, classifier bagged decision tree).

**Figure 6:**
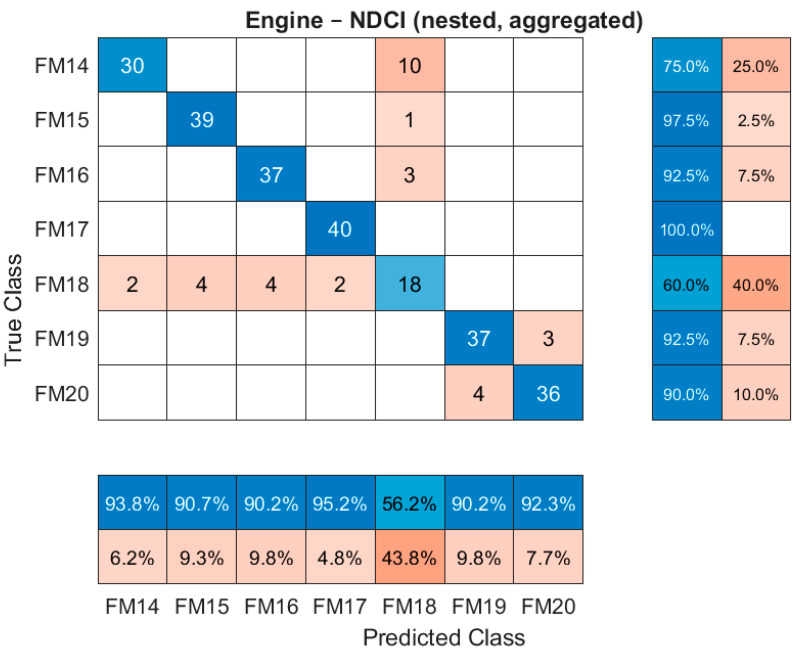
Confusion matrix for the engine subsystem using the NDCI-selected suite (nested, aggregated).

The aggregated confusion matrices from the nested protocol provide a detailed view of diagnostic performance. Figure 5 shows the confusion matrix for the EPS subsystem, and Figure 6 shows the matrix for the engine subsystem, both using the NDCI-selected suites.

For the EPS subsystem, unlike the engine, there are several areas of significant confusion. For example, both FM1 and FM2 are frequently classified as FM2 (33 instances each). Similarly, FM3 is often misclassified as FM5 (34 instances). This visualisation is crucial as it reveals not just the overall accuracy but also the specific failure modes that the current sensor suite struggles to disambiguate.

The engine confusion matrix visualises the classification performance using the NDCI-selected sensors. Each row represents the true fault class, and each column represents the predicted class. The strong diagonal, indicated by the dark blue cells with high counts (e.g., 39 for FM15, 40 for FM17), demonstrates a high true positive rate. Off-diagonal cells in orange indicate misclassifications, which are relatively infrequent, confirming the high overall accuracy reported in Table 4.

A repeated nested cross-validation pipeline is used for each subsystem independently (engine, fuel, ECS, EPS). In every outer fold, feature rankings are learned on the training partition only, and an inner K-fold loop evaluates a single, fixed classifier while adding sensors stepwise in rank order (top-1, top-2, …). The classifier is a class-weighted bagged decision-tree ensemble (weights inversely proportional to class frequency) and is kept identical for all ranking methods to ensure a fair comparison. At each step, the inner CV mean ± standard deviation of accuracy forms the stepwise curves; the selected number of sensors, k, for each ranking is chosen by a one-SE (standard error) rule. The model is then retrained on the outer-fold training data with the top k sensors and evaluated on the held-out test faults.

**Figure 7:**
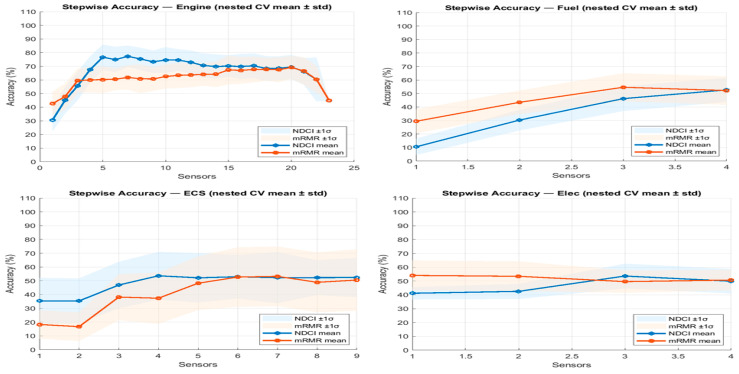
Stepwise accuracy for the engine subsystem (nested CV mean ± std): NDCI vs. mRMR.

Figure 7 consolidates the stepwise mean ± std curves for all four subsystems, comparing NDCI to mRMR under the same nested protocol. The engine and ECS panels indicate that NDCI achieves higher accuracy with fewer sensors, thereby improving sample efficiency. On the engine, accuracy rises rapidly and approaches ~75% by about five sensors with NDCI, whereas mRMR requires more sensors to achieve a similar level. For the fuel subsystem, mRMR holds a slight advantage; consistent with the smaller and less diverse fuel sensor set, where NDCI’s uniqueness term becomes more restrictive. The EPS panel is more competitive, with NDCI peaking slightly higher around three sensors.

**Figure 8:**
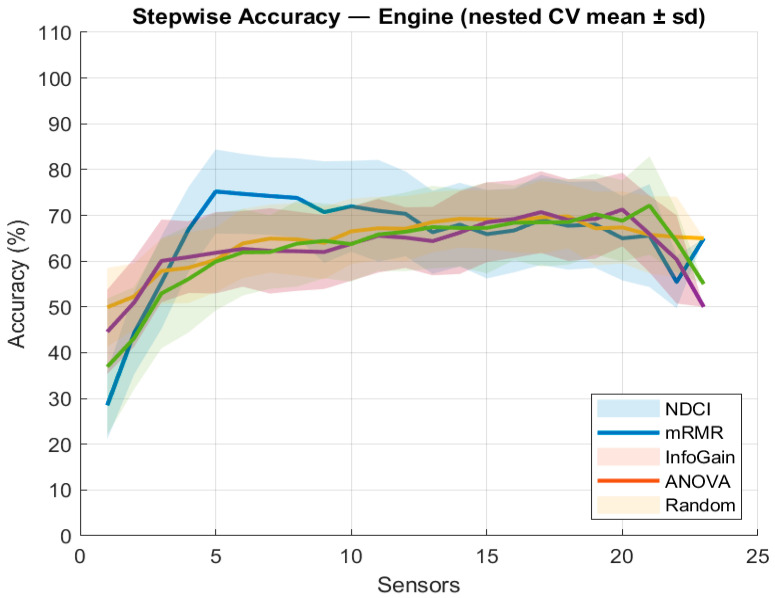
Stepwise accuracy for the engine subsystem (nested CV mean ± std—extra lighter coloured): NDCI, mRMR, Info-Gain, ANOVA, and Random.

To probe method sensitivity on a representative case, Figure 8 adds two additional ranking baselines on the engine subsystem. Info-Gain (mutual information) and ANOVA-F, together with a Random ranking obtained by randomly permuting the candidate list. Info-Gain and ANOVA closely track the mRMR family, while Random grows slowly and plateaus early, confirming that the gains stem from the ranking rather than the stepwise evaluation procedure. Across the range, NDCI remains the most sample-efficient, achieving high accuracy with a compact subset by jointly rewarding separation power, severity sensitivity, and uniqueness in ranking.

All stepwise plots in this subsection are per subsystem (the model considers Healthy plus that subsystem’s fault modes only) to isolate the effect of the ranking method and avoid cross-subsystem leakage. The cross-subsystem value is then exploited in the platform-level MOSOF study, where the final suite combines sensors from multiple subsystems.

**Figure 9:**
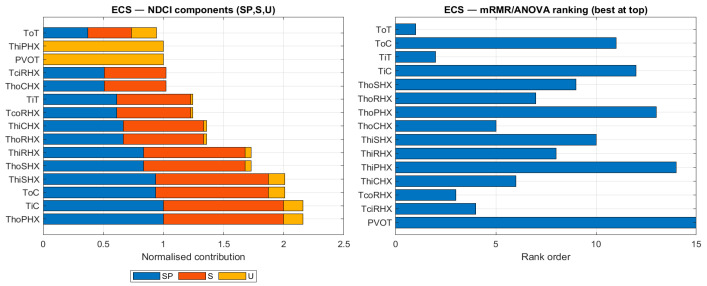
Ranking comparison for the ECS subsystem: NDCI components (SP, S, U) vs. mRMR ranking.

**Figure 10:**
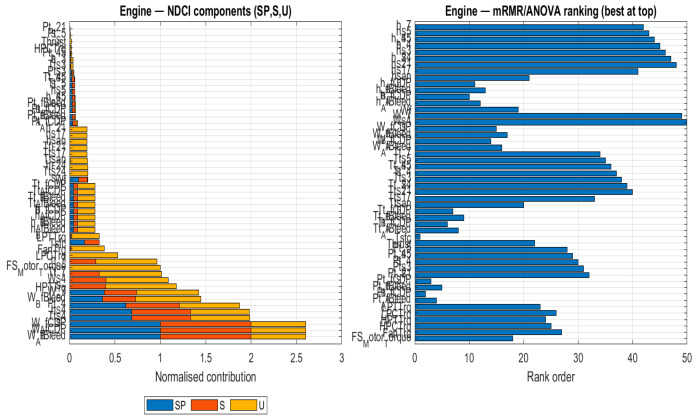
Ranking comparison for the engine subsystem: NDCI components (SP, S, U) vs. mRMR ranking.

**Figure 11:**
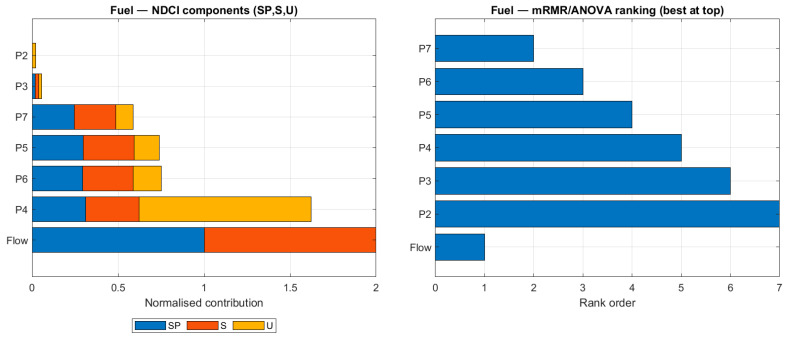
Ranking comparison for the fuel subsystem.

**Figure 12:**
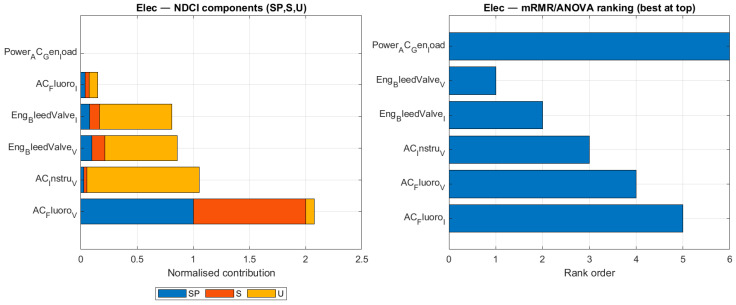
Ranking comparison for the EPS subsystem.

To understand the underlying reasons for the different sensor rankings, Figure 9, Figure 10, Figure 11 and Figure 12 compare the NDCI component breakdown with the mRMR rank order for each subsystem. Each figure has two panels for the same subsystem:

- Left panel—NDCI components (stacked bars).
  Each horizontal bar = one sensor. The bar is divided into three normalised NDCI components: SP (Separation Power), S (Severity Sensitivity), and U (Uniqueness). The total bar length is proportional to the composite NDCI (SP + S + U, i.e., higher is better).
  Ordering: sensors are sorted by composite NDCI, so “better” sensors appear lower in the plot. In other words, read the left panel from bottom to top: the bottom bars represent the highest NDCI sensors.
- Right panel—mRMR rank values (shorter is better).
  This panel shows only the rank order produced by the mRMR baseline. The bar length equals the numerical rank (1 = best, 2 = second-best, …).
  Important: The y-axis lists sensors alphabetically, not by rank. Consequently, rank 2 is not necessarily underneath rank 1. To interpret the panel, look at the number along the x-axis (bar length): shorter bars = better ranks.

This side-by-side view compares the two methods for ranked sensors: NDCI prioritises sensors with strong and balanced SP/S/U contributions, while mRMR focuses on relevance with redundancy control, which can favour different channels.

For example, in the engine (Figure 9), NDCI elevates sensors such as W_AfBleed and h_S4 because they exhibit clear fault separation, monotonic severity response, and low redundancy. In contrast, mRMR’s top-ranked items (short bars at the right) may cluster around a smaller set of correlated variables that are highly relevant but less unique.

Figure 9 provides a side-by-side comparison of sensor rankings for the ECS. The left panel shows the NDCI score for each sensor, broken down into its three components (SP, S, U). The right panel shows the rank order produced by mRMR. This visual comparison helps to explain why the methods select different sensors, highlighting NDCI’s emphasis on a balanced diagnostic contribution.

Similar to the previous figure, this plot (Figure 10) for the engine subsystem reveals the differing priorities of the two ranking methods. NDCI ranks the sensor readings based on its defined metric, whereas the mRMR ranking on the right is based on label relevance (mutual information) with redundancy control.

For the fuel subsystem, Figure 11 illustrates why NDCI struggled relative to mRMR. The NDCI component plot on the left shows that while flow has a high SP and S, the other sensors (P2–P7) have relatively low scores, especially when uniqueness is considered. This visualisation supports the conclusion that a lack of sensor diversity limited NDCI’s effectiveness in this specific case.

Figure 12 compares the sensor rankings for the EPS. It shows how NDCI prioritises sensors like AC_Fluoro_V and AC_Instru_V, which have a high uniqueness (yellow bar), meaning they provide information that is distinct from other available signals, a key factor for effective fault isolation in the EPS.

The final accuracies for the NDCI-selected suites across all subsystems are visualised in Figure 12.

**Figure 13:**
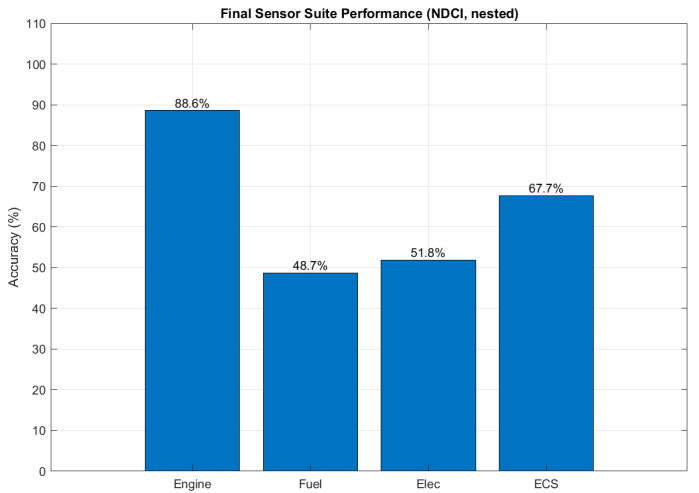
Final sensor-suite accuracy comparison (best-k, nested).

The bar chart in Figure 13 summarises the peak diagnostic performance (balanced accuracy) achieved by the NDCI-selected sensor suites for each subsystem under the robust nested validation protocol. The engine subsystem clearly shows the highest diagnostic accuracy at 88.6%, followed by the ECS at 67.7%. The lower performance for the EPS (51.8%) and fuel (48.7%) subsystems indicates a greater intrinsic difficulty in diagnosing their respective fault modes with the available instrumentation.

**Figure 14:**
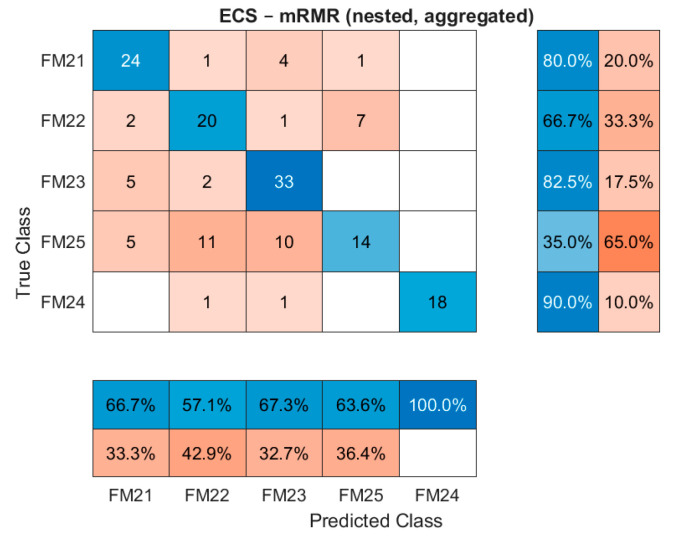
ECS confusion matrix for the mRMR suite (nested, aggregated).

**Figure 15:**
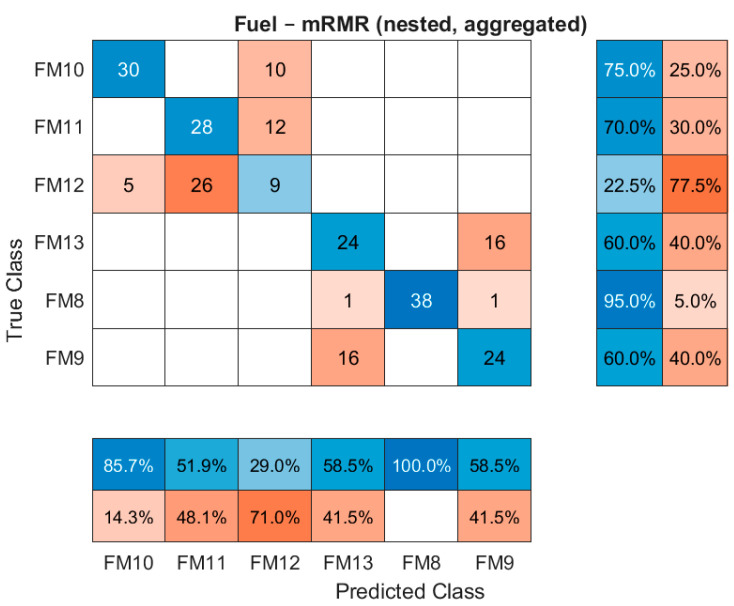
Fuel confusion matrix for the mRMR suite (nested, aggregated).

For completeness, the confusion matrices for the cases where mRMR competes with (Figure 14) and marginally outperforms NDCI (Figure 15) are presented.

Figure 14 shows the performance of the mRMR-selected sensor suite for the ECS. While overall accuracy is lower than that for NDCI, this view allows for a direct comparison of which specific fault modes mRMR handles effectively.

This confusion matrix in Figure 15 details the performance of the mRMR-selected suite for the fuel subsystem, where it achieved a slightly higher balanced accuracy than NDCI. It highlights the specific classification strengths and weaknesses of the mRMR approach in a sensor-constrained environment.

## 4. Airline-Centric MOSOF Trade-Off Study

**Figure 16:**
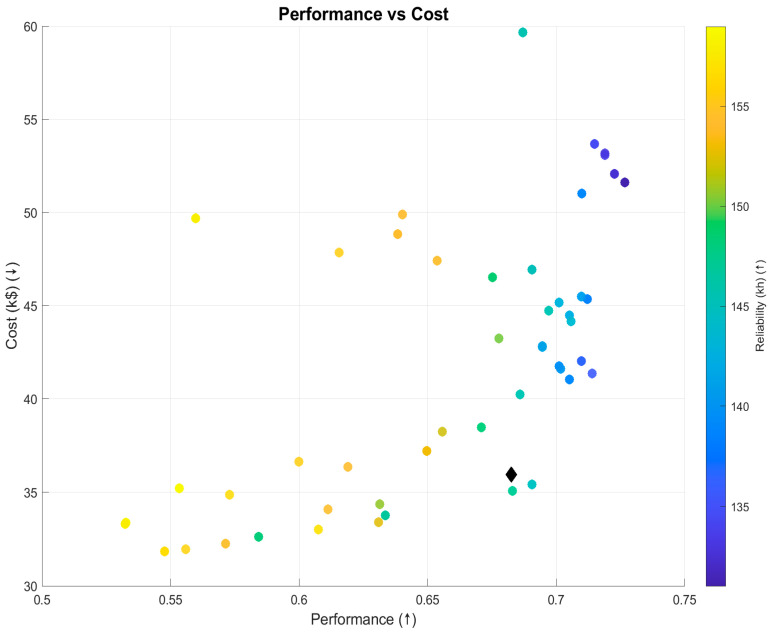
Feasible Pareto set (performance vs. cost; colour = reliability).

**Figure 17:**
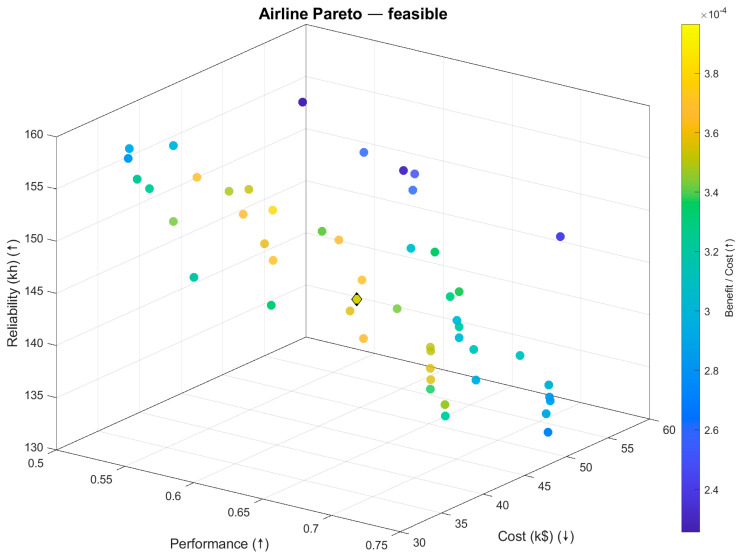
Three-dimensional Pareto front with benefit-to-cost colouring; the knee solution is marked.

MOSOF was executed to find a globally optimised sensor suite for an airline stakeholder whose objectives are discussed and evaluated in the previous paper on the ECS case. The resulting Pareto front, containing all non-dominated solutions, is visualised in Figure 16 and Figure 17. These plots illustrate the trade-off surface between performance, cost, and reliability.

Figure 16 shows the projection of the tri-objective Pareto set onto performance vs. cost, with reliability encoded by colour. Because this is a projection, it does not imply a monotonic relation between cost and performance. In fact, the cloud exhibits three clear regimes:

- A flat, budget floor around USD32–36k where additional spend yields little performance change;
- A moderate-slope region up to ≈0.68 performance;
- A steep, diminishing-returns region beyond ≈0.69 where sizeable cost increases buy only small gains.

At ≈0.70 performance, several designs span USD40–USD55k with similar performance. This is expected: these points differ along the reliability dimension (colour) and in their sensor mix. Some higher-cost suites utilise pricier channels or incorporate redundancy that enhances or maintains reliability, while others compromise reliability for marginal performance improvements. The black diamond marks the knee—the solution on the front with the largest normalised gain in performance (and reliability) per unit cost relative to its immediate neighbours (the “elbow” of the curve in this 2D view).

Figure 17 plots the same non-dominated set in three dimensions: performance (x ↑ maximised), cost (y ↓ minimised), and reliability (z ↑ maximised). This view adds two things that the 2D projection cannot show:

- Why do points with similar performance–cost differ? The third axis reveals their reliability separation. For example, the designs clustered near 0.70 performance in Figure 15 occupy different heights in Figure 16 (corresponding to different reliability levels), which explains the wide cost band seen in the projection.
- Where the efficient “ridge” lies in 3D, the front forms a curved surface; moving toward higher performance can either lift reliability (good) or flatten/drop it (undesirable). Seeing this surface helps identify regions where small cost increases improve both performance and reliability, versus areas where one improves at the expense of the other.

The colour in Figure 17 shows a benefit-to-cost ratio (a normalised combination of performance and reliability divided by cost), purely for visual emphasis. Brighter colours flag suites that deliver more capability per dollar; they are not used to define Pareto optimality. The yellow diamond represents the knee solution (same design as the black diamond in Figure 16), chosen at the highest-curvature region of the front after objective normalisation; it is the point beyond which the incremental cost yields progressively smaller joint gains.

**Figure 18:**
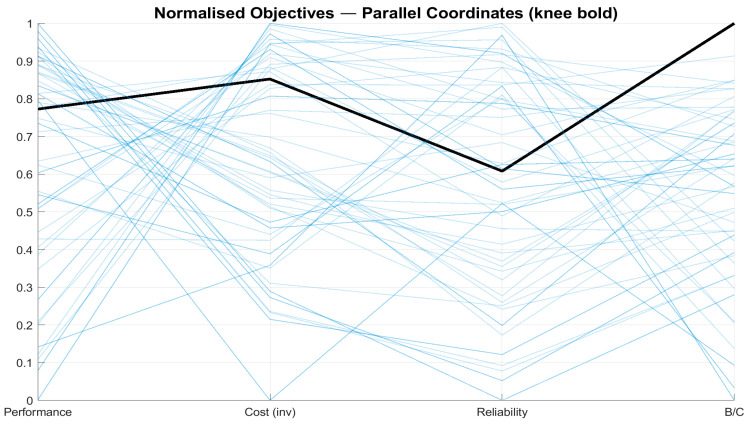
Parallel-coordinates plot of normalised objectives; the knee solution is bolded.

Figure 18, a parallel coordinates plot, enables the comparison of all Pareto-optimal solutions across the four normalised objectives. Each light blue line is a single sensor suite. The bold black line represents the knee solution. It clearly demonstrates that this solution achieves a high level of performance, reliability, and benefit-to-cost, while requiring only a moderate compromise on cost (shown as inverted).

The identified knee solution provides a concrete recommendation. It consists of 12 sensors: 5 from the engine, 2 from the fuel system, 2 from the EPS, and 3 from the ECS. This suite achieves a normalised performance score of 0.69 at a cost of approximately USD36k, with a combined reliability of 145 kh MTBF, as summarised in Table 5.

From subsystem validation to the platform recommendation (how Table 5 is obtained):

1. Per-subsystem evidence (Section 3.1 and Section 3.2).
  Sensors are ranked within each subsystem using the NDCI values; these rankings provide the diagnostic value of candidate sensors.
2. Platform design space and objectives.
  A feasible catalogue of multi-subsystem SENSORS ARTRIBUTIONS is hypothetically generated under integration rules and subsystem quotas. Each suite is scored on the three primary objectives: performance (↑ NDCI-based diagnostic score aggregated over selected sensors), cost (↓ purchase + integration), and reliability (↑ suite MTBF as defined below). A derived view, benefit-to-cost (↑ a normalised combination of performance and reliability per dollar), is used only for visual triage and does not define Pareto optimality.

Sensor-attribute catalogue (inputs and ranges used for optimisation):

The optimisation uses a curated catalogue of 96 candidate sensors spanning four subsystems. Across the catalogue:

- Costs range from USD240–USD12.5k (medians by subsystem: engine USD1.55k, fuel USD1.16k, EPS USD0.65k, ECS USD0.65k).
- MTBFs (MTBFsuite = 1/∑*i*(1/MTBF*i*)) span 88–350 kh (medians by subsystem: EPS 177 kh, ECS 151 kh, engine 148 kh, fuel 119 kh; harmonic-mean MTBFs: EPS 184 kh, ECS 159 kh, engine 144 kh, fuel 120 kh).
- By sensor family, typical cost/MTBF ranges are:
  - Flow: USD4.0–12.5k, 88–150 kh
  - Torque: USD2.54–6.48k, 91–152 kh
  - Pressure: USD0.88–2.38k, 105–193 kh
  - Temperature: USD0.46–1.19k, 127–219 kh
  - Thermodynamic property: USD0.81–1.85k, 101–187 kh
  - Electrical (voltage/current/power): USD0.24–1.26k, 161–232 kh
  - Computed metrics (e.g., thrust, TSFC): USD300, 350 kh

These ranges (detailed list of the sensor attributes is shared with the code bundle [20]) explain the patterns in the Pareto plots: flow/torque channels tend to be costly and less reliable, temperature/electrical channels are cost-efficient with higher MTBF, and engine reliability is quantitatively close to ECS (medians 148 kh vs. 151 kh), so “EPS/ECS most reliable” should be read as small, catalogue-level advantages, not categorical separation.

**Figure 19:**
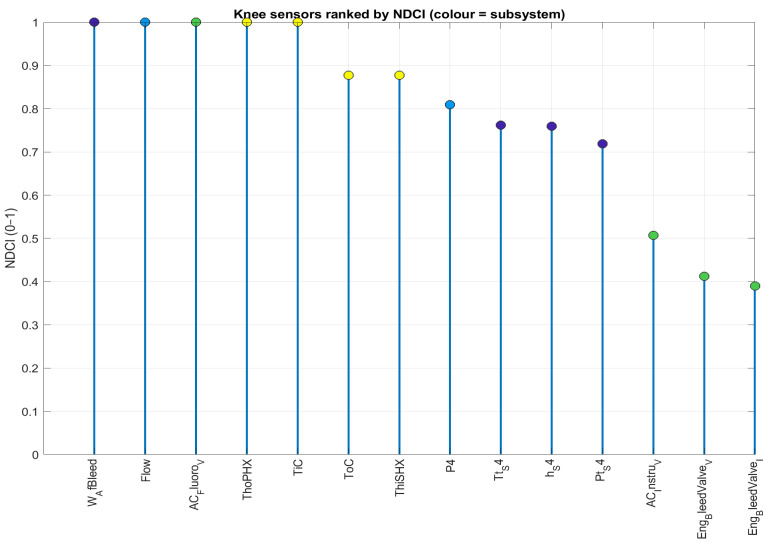
The sensors comprising the knee solution, ranked by their NDCI score.

**Figure 20:**
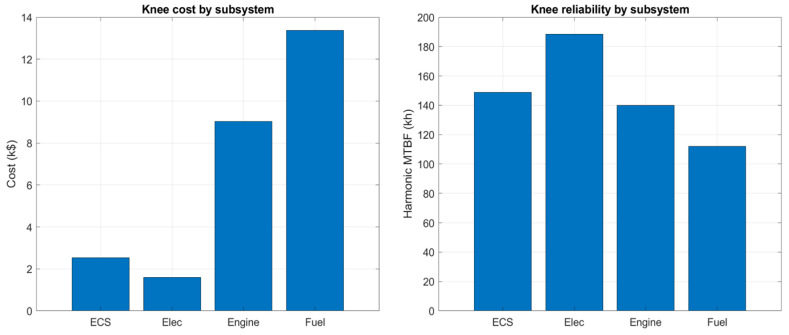
Cost and reliability breakdown of the knee suite by subsystem.

3. Pareto filtering and knee selection.
  Non-dominated suites form the Pareto set (Figure 16 and Figure 17). A knee is chosen at the point of highest trade-off efficiency in normalised objective space (largest local curvature/smallest distance to the utopia corner among neighbours). That single design is summarised in Table 5 and detailed in Figure 19 and Figure 20.

The lollipop plot in Figure 19 ranks the individual sensors included in the knee solution by their NDCI score. The height of each stem represents the diagnostic value of that sensor. The colours indicate the source subsystem. The mix reflects the catalogue’s economics (with educated guesses): ECS temperature and electrical families provide cost-efficient, reliable coverage, while a limited number of engine/fuel flow channels supply targeted separation despite higher cost and lower MTBF.

These bar charts in Figure 20 detail the composition of the knee solution. The left panel accumulates cost by subsystem for the twelve selected sensors; higher spending is associated with engine/fuel flow families (catalogue costs in the USD4–12.5k band), whereas ECS temperature and EPS electrical measurements contribute at lower unit cost.

The right panel reports a descriptive harmonic-mean MTBF for the sensors drawn from each subsystem in the knee. Catalogue medians indicate EPS ≈ 177 kh, ECS ≈ 151 kh, and engine ≈ 148 kh (engine is close to ECS), so differences are modest. The suite-level reliability is governed primarily by the lowest-MTBF elements included (typically flow/torque), which explains the overall ≈ 145 kh value in Table 5. Please note that the complete sensor-attribute table (including per-sensor cost and MTBF values) is provided with the code bundle [20] to ensure reproducibility without overloading the main text.

## 5. Discussion

The results show that a domain-specific ranking metric, NDCI, yields more informative and compact diagnostic suites than generic relevance-based methods, particularly mRMR, ANOVA, and Information Gain. The advantage is most visible in engine and EPS subsystems, where sensor counts are higher: NDCI’s uniqueness term suppresses redundant channels within a cluster, so the selected set spans complementary loci of the physical process rather than multiple views of the same point.

The knee suite reflects these macro couplings: a small number of high-leverage flow/enthalpy channels (fuel and bleed-air) are combined with temperature chain and electrical measurements that respond downstream. Generic relevance methods (e.g., mRMR/ANOVA) tend to cluster within a single subsystem’s highly correlated family (e.g., multiple adjacent temperatures). In contrast, NDCI’s uniqueness term distributes picks along the physical pathway—from cause to propagation—yielding better isolation with fewer sensors.

The Pareto front provides a map rather than a single prescription. A cost-constrained operator can select within the USD32–36k band, where performance is relatively flat; a certification-focused OEM can move toward points with higher reliability at additional cost; maintenance organisations can choose the lowest-cost feasible suites and accept a small reduction in detection/isolation capability. The 3D view clarifies why designs with similar performance and cost separate in practice, reliability differs, and helps avoid choices that raise performance while inadvertently depressing reliability.

The dataset is simulated; validation on rig or fleet data is essential to capture noise, drift, and maintenance effects. Consequently, the “clean” nature of the simulation data may yield slightly optimistic NDCI scores compared to real-world operations, where signal-to-noise ratios are higher. Cost and MTBF inputs come from a curated attribute catalogue (vendor figures where available, otherwise documented class averages); suite reliability is computed as a series-equivalent MTBF (reciprocal of summed failure rates), which appropriately penalises the weakest elements but ignores shared-cause failures. Extending the redundancy model beyond linear correlation, injecting prognostic value as an additional objective, and stress-testing robustness to data missingness are natural next steps.

The MOSOF results translate these technical findings into actionable, stakeholder-centric intelligence. The Pareto front is not just a collection of solutions but a strategic decision-making tool. An airline can use the recommended knee point to implement a cost-effective diagnostic upgrade. An Original Equipment Manufacturer (OEM), who may prioritise system reliability for certification purposes, could select a different point on the Pareto front that offers a higher MTBF at the cost of a few additional sensors. An MRO provider might prioritise a suite with the absolute lowest cost, accepting a small reduction in diagnostic accuracy. By delivering the entire Pareto front, MOSOF empowers each stakeholder to make informed decisions based on their unique constraints and priorities without needing to re-run the entire optimisation analysis.

In sum, the combination of platform-level symptom evidence, properly nested subsystem validation, and multi-objective optimisation explains both the numerical results and their physical meaning: the preferred suites are those that trace the distinguished faults identified with the NDCI score while meeting cost and reliability constraints.

## 6. Conclusions

This paper presents a refined and rigorously validated framework for multi-objective sensor optimisation, built on the foundation of the diagnostically aware NDCI metric. The comprehensive, cross-subsystem evaluation demonstrated that the NDCI-driven approach consistently produces more compact and effective sensor suites for complex diagnostic tasks than conventional methods like mRMR. The use of a nested cross-validation protocol ensures that the reported performance metrics are robust and reliable.

By integrating NDCI within the MOSOF, a powerful tool has been created that translates complex engineering trade-offs into a clear set of Pareto-optimal solutions. The application to an airline case study yielded a practical, cost-effective sensor suite that leverages synergistic information from across multiple subsystems. This work represents a significant step towards a more systematic, evidence-based methodology for designing the next generation of IVHM systems, enabling a shift from reactive to predictive maintenance and enhancing the safety and efficiency of complex machinery.

Designing diagnostic sensor suites for integrated aircraft requires balancing performance, cost, and reliability while capturing cross-subsystem fault propagation. The study establishes a complete and reproducible pathway for this task by combining (i) platform-level vectors to expose macro effects, (ii) rigorous, nested per-subsystem validation to obtain unbiased performance estimates of NDCI, and (iii) a MOSOF that delivers stakeholder-ready trade-offs.

Key findings are as follows.

- Diagnostic ranking: Across subsystems, the NDCI (which scores sensors by separation power, severity sensitivity, and uniqueness) consistently produces more compact and practical suites than relevance-based baselines. Under the nested protocol, NDCI outperforms mRMR on engine (balanced accuracy 0.886 vs. 0.690) and ECS (0.677 vs. 0.520), is comparable on EPS (0.518 vs. 0.510), and concedes a small margin on fuel (0.487 vs. 0.530), where limited sensor diversity constrains uniqueness. These results align with the stepwise inner CV curves, which show that NDCI reaches target accuracy with fewer sensors, indicating improved sample efficiency.
- Cross-subsystem evidence: Evaluation on PSV and severity sweeps of fault modes reveals physically plausible macro pathways; for example, fuel to engine via combustion signatures, engine bleed-air to ECS along the heat-exchanger temperature chain, and ECS to EPS via load changes were all visible on the associated PSVs. FM5’s PSV is presented in Section 3.1 for illustrative purposes; other PSVs can be obtained from the code bundle provided in the references. NDCI’s uniqueness term disperses selections along these pathways, avoiding clusters of near-duplicate measurements. This explains the higher isolation capability with compact suites.
- Stakeholder trade-offs and the Pareto front: Multi-objective optimisation over performance↑—cost↓—reliability↑ yields a feasible Pareto set with clear regimes: a low-cost, flat-performance floor; a moderate-slope region; and a diminishing-returns region near 0.69–0.71 performance. The knee solution—identified by maximum local curvature in normalised objective space—comprises 12 sensors (Engine 5, Fuel 2, EPS 2, ECS 3) delivering ≈ 0.69 performance at ≈USD36k with ≈145 kh suite MTBF. The 3D Pareto view clarifies why designs with similar performance and cost can differ materially (reliability is the key factor that separates them), supporting informed choices for airlines, OEMs, or MROs.
- Reliability treatment: Suite-level reliability is computed as a series-equivalent MTBF (reciprocal of summed failure rates), which appropriately penalises the weakest elements. Catalogue medians show only modest differences among subsystems (Engine close to ECS), so overall suite reliability is driven by the specific sensor families selected (e.g., flow/torque tend to be costlier and less reliable than temperature/electrical).

The integrated NDCI–MOSOF workflow translates raw multi-sensor data into transparent, defensible evidence for IVHM design. It avoids optimistic bias through proper nesting, exposes the physical meaning of selections via component-wise ranking visuals, and delivers a portfolio of optimal suites rather than a single prescription, enabling stakeholder-specific decisions without re-running the complete analysis.

Limitations and future work. Findings are derived from a high-fidelity virtual aircraft and curated cost/MTBF catalogues. Validation on rig and in-service fleet data is required to account for noise, drift, maintenance actions, and shared-cause failures. Future extensions include (i) richer redundancy models beyond linear correlation, (ii) incorporation of prognostic value (e.g., RUL) as an additional objective, (iii) robustness to missing data and varying mission profiles, and (iv) uncertainty-aware optimisation of cost and reliability inputs. Also, safety-critical hardware redundancy would need to be added as hard constraints for certification-ready designs.

Overall conclusion. A diagnostically aware ranking coupled with principled, multi-objective optimisation provides a traceable, platform-level route to sensor suites that capture cross-subsystem effects while respecting economic and reliability constraints. The approach is readily transferable to other complex assets and supports model-based safety and certification processes by delivering reproducible, decision-grade artefacts—from stepwise validation to Pareto fronts and a clearly justified knee recommendation.

## Figures and Tables

**Table 1:** Fault mode descriptions.

EPS	“FM1”	AC Motor Fault (FS Motor)
EPS	“FM2”	FS Nozzle Switch Open
EPS	“FM3”	FS Valve Switch Open
EPS	“FM4”	Engine Bleed Valve Switch Open
EPS	“FM5”	ECS TCV Switch Open
EPS	“FM6”	AC Lamp Instru Switch Open
EPS	“FM7”	AC Lamp Fluoro Switch Open
FS	“FM8”	Pump External Leakage (DPV1)
FS	“FM9”	Pump Internal Leakage (DPV2)
FS	“FM10”	FOHE Clogging (DPV3)
FS	“FM11”	FOHE Leakage (DPV4)
FS	“FM12”	Fuel Nozzle Clogging (DPV5)
FS	“FM13”	Reduced Pump RPM
ENG	“FM14”	LPT Blade Broken
ENG	“FM15”	LPC Fouling
ENG	“FM16”	HPT Blade Broken
ENG	“FM17”	HPC Contamination
ENG	“FM18”	Fan FOD
ENG	“FM19”	Bleed Valve Angle
ENG	“FM20”	CDP Leak
ECS	“FM21”	Primary Heat Exchanger (PHX) Fouling
ECS	“FM22”	PHX—Blockage of Cold Mass Flow
ECS	“FM23”	Secondary Heat Exchanger (SHX) Fouling
ECS	“FM24”	Air Cycle Machine (ACM) Mechanical Efficiency
ECS	“FM25”	RAM Mass Flow Blockage

**Table 2:** Best-performed classifier comparison per subsystem.

Subsystem	Task	Best Classifier
Engine	Detection	Bag
Engine	Isolation	Bag
Fuel	Detection	Bag
Fuel	Isolation	nbKernel
EPS (Elec)	Detection	Bag
EPS (Elec)	Isolation	svmRBF
ECS	Detection	Bag
ECS	Isolation	Bag

**Table 3:** Baseline cross-validation results for each subsystem.

Subsystem	Method	Balanced Accuracy	Sensors Used
Engine	NDCI	0.926	9
Engine	mRMR	0.778	12
Fuel	NDCI	0.667	5
Fuel	mRMR	0.583	7
EPS	NDCI	0.731	8
EPS	mRMR	0.346	12
ECS	NDCI	0.812	6
ECS	mRMR	0.812	8

**Table 4:** Nested, aggregated cross-validation results.

Subsystem	Method	Balanced Accuracy	Sensors Used
Engine	NDCI	0.886	10
Engine	mRMR	0.690	12
Fuel	NDCI	0.487	5
Fuel	mRMR	0.530	5
EPS	NDCI	0.518	7
EPS	mRMR	0.510	8
ECS	NDCI	0.677	6
ECS	mRMR	0.520	7

**Table 5:** Composition and objective values for the knee point obtained by multi-objective optimisation.

Objective	Knee Value	Description
Diagnostic Performance	≈0.69	Normalised NDCI-based score of the selected suite
Cost	≈USD36k	Sum of sensor purchase and integration costs
Reliability	≈145 kh	Harmonic mean of sensor MTBFs
Sensors per Subsystem	Engine 5, Fuel 2, EPS 2, ECS 3	Composition of the knee suite

## Data Availability

All data and code that support the findings of this study are provided at GitHub repository, and its link is provided in the references.

## Abbreviations

OEM	Original Equipment Manufacturer
MRO	Maintenance Repair Overhaul
MTBF	Mean Time Between Failures
VAM	Virtual Aircraft Model
EPS	Electrical Power System
ECS	Environmental Control System
mRMR	minimum Redundancy–maximum Relevance
MOSOF	Multi-Objective Sensor Optimisation Framework
NDCI	Normalised Diagnostic Contribution Index
IVHM	Integrated Vehicle Health Management
